# Supplementary material for: A family of linear plasmid phages that detect a quorum-sensing autoinducer exists in multiple bacterial species
Source: bioRxiv. 2025 Jul 30:2025.07.30.667625. Preprint. [Version 1] doi: 10.1101/2025.07.30.667625 (PMC12324486; doi:10.1101/2025.07.30.667625)
Supplement: 1 [file NIHPP2025.07.30.667625v1-supplement-1.pdf]

bioRxiv preprint doi: https://doi.org/10.1101/2025.07.30.667625; this version posted July 30, 2025. The copyright holder for this preprint (which was not certified by peer review) is the author/funder, who has granted bioRxiv a license to display the preprint in perpetuity. It is made available under aCC-BY 4.0 International license.

SUPPLEMENTARY MATERIAL

A family of linear plasmid phages that detect a quorum-sensing autoinducer exists in multiple bacterial species.

Santoriello, FJ & Bassler, BL

Figure S1. Hapunaviruses are subdivided into VP882-like phages and HAP-1-like phages.

Figure S2. VqmAφ and cI vary across VP882-like phages, whereas Qtip is highly conserved.

Figure S3. LuxO-OpaR(LuxR) and VqmAR quorum-sensing proteins are restricted to vibrios, while the autoinducer synthases Tdh and LuxS are conserved across genera.

Table S1. Genome metadata for VP882-like linear plasmid phages.

Table S2. Strains and plasmids used in this study.

Table S3. Primers and synthetic DNA fragments used in this study.

Supplementary Dataset 1. Identifiers for all genomes used in this study.

Supplementary Dataset 2. vConTACT assigned viral clusters for all phage genomes.

Supplemental References

bioRxiv preprint doi: https://doi.org/10.1101/2025.07.30.667625; this version posted July 30, 2025. The copyright holder for this preprint (which was not certified by peer review) is the author/funder, who has granted bioRxiv a license to display the preprint in perpetuity. It is made available under aCC-BY 4.0 International license.

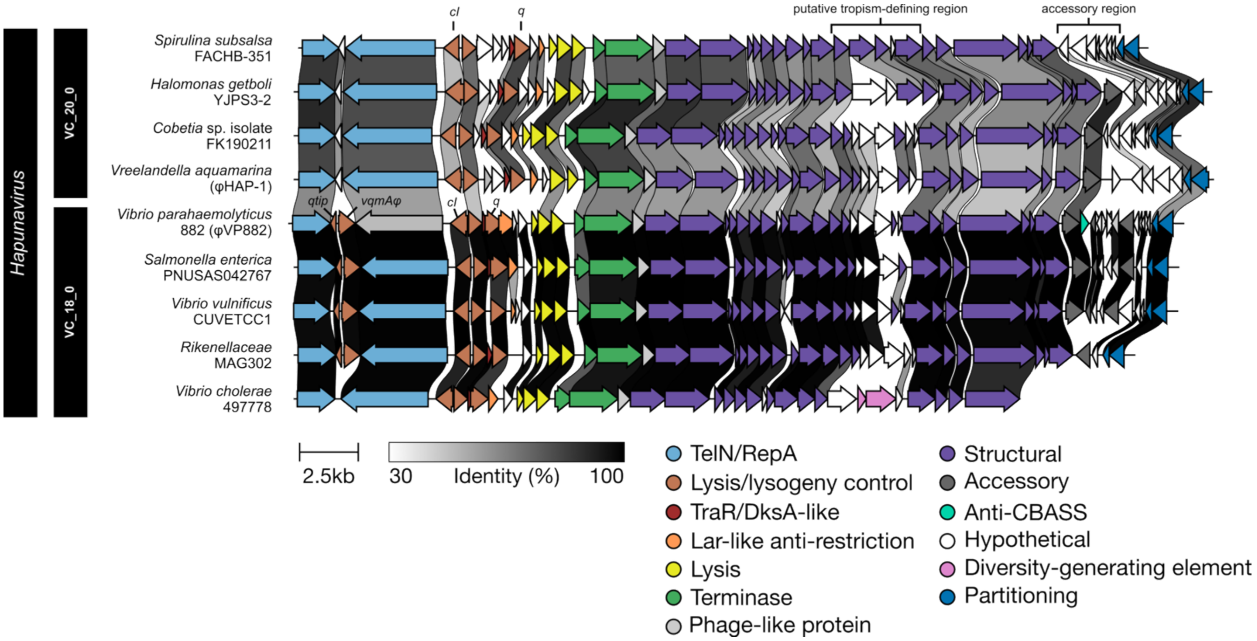

Fig S1. Hapunaviruses are subdivided into VP882-like phages and HAP-1-like phages. Genome synteny of HAP-1-like (VC_20_0) and VP882-like (VC_18_0) linear plasmid phages. Host species and strain are provided on the left. Arrows represent genes colored according to their annotated functions. Gene homologs in neighboring sequences are connected by shaded links. The shading represents the % identity between the amino acid sequences of the proteins encoded by the homologous genes. The absence of a link indicates less than 30% amino acid identity between proteins encoded by neighboring genes or the absence of a homolog in the neighbor.

bioRxiv preprint doi: https://doi.org/10.1101/2025.07.30.667625; this version posted July 30, 2025. The copyright holder for this preprint (which was not certified by peer review) is the author/funder, who has granted bioRxiv a license to display the preprint in perpetuity. It is made available under aCC-BY 4.0 International license.

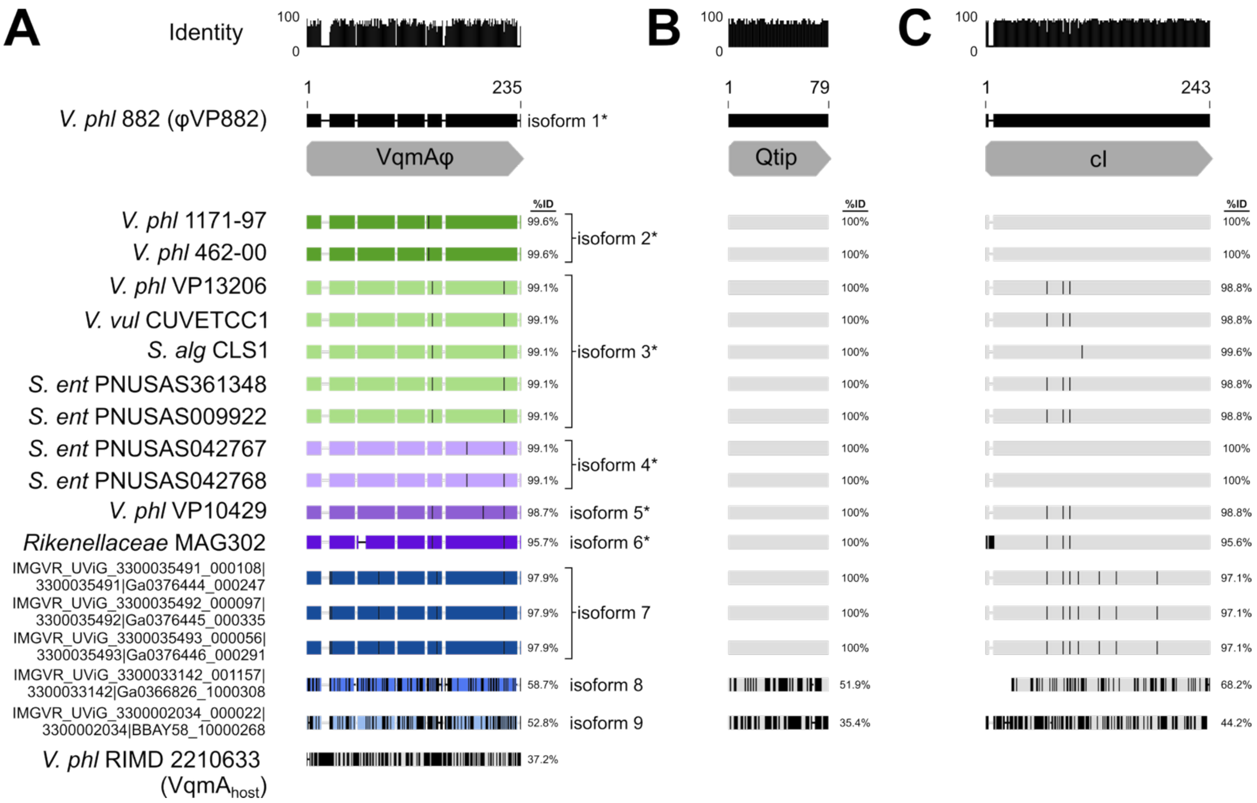

Fig S2. VqmAφ and cI vary across VP882-like phages, whereas Qtip is highly conserved. Pairwise amino acid alignments of (A) VqmAφ, (B) Qtip, and (C) cI proteins from the 17 VP882-like phages with intact *vqmAφ-qtip* modules (see Fig. 2A of the main text). Host species and strain are provided on the left (*V. phl* = *Vibrio parahaemolyticus*, *V. vul* = *Vibrio vulnificus*, *V. cho* = *Vibrio cholerae*, *S. ent* = *Salmonella enterica*, *S. alg* = *Shewanella algeae*). Sequences designated IMGVR were collected from metagenomic data and thus, do not have associated host strains. Horizontal bars represent the homologous protein sequences, and black vertical lines within the bars represent amino acid differences relative to the VqmAφ, Qtip, and cI proteins from φVP882. Thin black horizontal lines denote gaps in sequences compared to the reference sequence. Thin gray horizontal lines denote gaps in sequences compared to any sequence other than the reference sequence. In (A), VqmAφ proteins are colored and labeled by isoform. Isoforms marked with asterisks were used in assays in the main text. The $VqmA_{host}$ protein sequence from *V. parahaemolyticus* str RIMD 2210633 was included to demonstrate that while VqmAφ isoforms 8 and 9 vary most among phage VqmAφ homologs, they share greater identity with VqmAφ than the identity shared between $VqmA_{host}$ and VqmAφ. This finding supports the logic that all the VqmAφ isoforms shown should be considered VqmAφ rather than some other transcription factor.

bioRxiv preprint doi: https://doi.org/10.1101/2025.07.30.667625; this version posted July 30, 2025. The copyright holder for this preprint (which was not certified by peer review) is the author/funder, who has granted bioRxiv a license to display the preprint in perpetuity. It is made available under aCC-BY 4.0 International license.

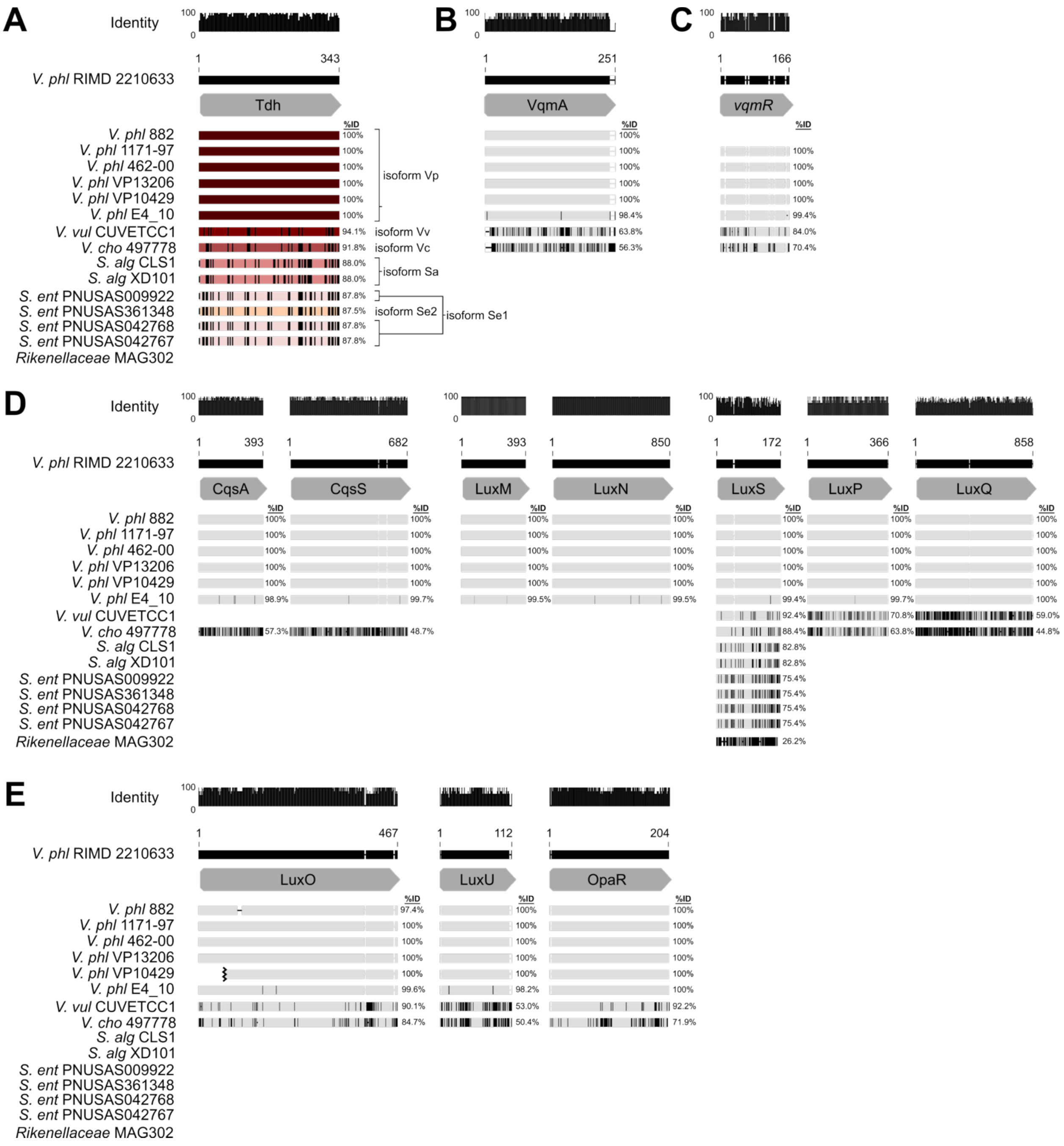

Fig S3. LuxO-OpaR(LuxR) and VqmAR quorum-sensing proteins are restricted to vibrios, while the autoinducer synthases Tdh and LuxS are conserved across genera. Pairwise alignments of (A) Tdh, (B) $VqmA_{host}$, (C) *vqmR*, (D) the autoinducer synthase and receptor proteins associated with the LuxO-OpaR pathway, and (E) LuxO, LuxU, and OpaR from 15 host strains harboring VP882-like phages (see Fig. 2A of the main text). (A-E) Host species and strain are provided on the left (*V. phl* = *Vibrio parahaemolyticus*, *V. vul* = *Vibrio vulnificus*, *V. cho* = *Vibrio cholerae*, *S. ent* = *Salmonella enterica*, *S. alg* = *Shewanella algeae*). Horizontal bars represent the homologous (A,B,D,E) protein or (C) nucleotide sequences, and black vertical lines within the bars represent (A,B,D,E) amino acid or (C) nucleotide differences relative to the corresponding sequences from the *V. parahaemolyticus* reference strain RIMD 2210633. Thin black horizontal

bioRxiv preprint doi: https://doi.org/10.1101/2025.07.30.667625; this version posted July 30, 2025. The copyright holder for this preprint (which was not certified by peer review) is the author/funder, who has granted bioRxiv a license to display the preprint in perpetuity. It is made available under aCC-BY 4.0 International license.

lines denote gaps in sequences compared to the reference sequence. Thin gray horizontal lines denote gaps in sequences compared to any sequence other than the reference sequence. (A) Tdh proteins colored and labeled by isoform. (E) The black jagged mark in the *V. phl* VP10429 sequence denotes the end of the contig.

bioRxiv preprint doi: https://doi.org/10.1101/2025.07.30.667625; this version posted July 30, 2025. The copyright holder for this preprint (which was not certified by peer review) is the author/funder, who has granted bioRxiv a license to display the preprint in perpetuity. It is made available under aCC-BY 4.0 International license.

65 **Table S1. Genome metadata for VP882-like linear plasmid phages.**

Host Strain	vConTACT2 Viral Cluster	Isolation Date	Isolation Location	Source
Vibrio parahaemolyticus 882 (1)	VC_18_0	unknown (published 2009)	Taiwan	unknown
Vibrio parahaemolyticus 1171-97	VC_18_0	1997	Peru: Moquegua	clinical
Vibrio parahaemolyticus 462-00	VC_18_0	2000	Peru: Lima	clinical
**Vibrio parahaemolyticus* 461-00	n/a	2000	Peru: Lima	clinical
***Vibrio parahaemolyticus* E4_10	n/a	2014	China: Zhejiang	fish
Vibrio parahaemolyticus str VP13026	VC_18_0	2013	China: Shenzhen	clinical
Vibrio parahaemolyticus str VP10429	VC_18_0	2010	China: Shenzhen	clinical
Vibrio vulnificus str CUVETCC1	VC_18_0	2021	Thailand: Chachoengsao	fish
Vibrio cholerae str 497778	VC_18_0	2017	unknown	clinical
Salmonella enterica str PNUSAS042767	VC_18_0	2018	USA	clinical
Salmonella enterica str PNUSAS042768	VC_18_0	2018	USA	clinical
Salmonella enterica str PNUSAS361348	VC_18_0	2023	USA	clinical
Salmonella enterica str PNUSAS009922	VC_18_0	2017	USA	clinical
Shewanella algae str XD101	VC_18_0	2023	China: Sanya	seawater
***Shewanella algae* str CLS1	n/a	2014	Taiwan	clinical
Rikenellaceae bacterium MAG302	VC_18_0	2017	New Zealand: Little Barrier Island, Auckland	fish gut metagenome
IMGVR_UViG_3300011259_000019, 3300011259, Ga0151662_1001257	VC_18_0	2015	Japan: Japan Sea near Toyama Prefecture	marine sediment
IMGVR_UViG_3300035491_000108, 3300035491, Ga0376444_000247	VC_18_0	2007	Trinidad and Tobago: La Brea, Pitch Lake	asphalt lake
IMGVR_UViG_3300035492_000097, 3300035492, Ga0376445_000335	VC_18_0	2007	Trinidad and Tobago: La Brea, Pitch Lake	asphalt lake

bioRxiv preprint doi: https://doi.org/10.1101/2025.07.30.667625; this version posted July 30, 2025. The copyright holder for this preprint (which was not certified by peer review) is the author/funder, who has granted bioRxiv a license to display the preprint in perpetuity. It is made available under aCC-BY 4.0 International license.

*IMGVR_UViG_3300035494_000080, 3300035494, Ga0376447_000328	n/a	2007	Trinidad and Tobago: La Brea, Pitch Lake	asphalt lake
IMGVR_UViG_3300035493_000056, 3300035493, Ga0376446_000291	VC_18_0	2007	Trinidad and Tobago: La Brea, Pitch Lake	asphalt lake
IMGVR_UViG_3300042259_000043, 3300042259, Ga0451649_000837	VC_18_0	2019	USA: Denver, Colorado	industrial wastewater
IMGVR_UViG_3300042267_000060, 3300042267, Ga0451650_000569	VC_18_0	2019	USA: Denver, Colorado	industrial wastewater
*IMGVR_UViG_3300042269_000097, 3300042269, Ga0451652_000962	n/a	2019	USA: Denver, Colorado	industrial wastewater
IMGVR_UViG_3300033142_001157, 3300033142, Ga0366826_1000308	VC_18_0	unknown (added to database 2019)	Mexico: Gulf of California	marine sediment
[†]IMGVR_UViG_3300002034_000022, 3300002034, BBAY58_10000268	Outlier	unknown (added to database 2013)	Australia: Sydney, Bare Island	red algae

* Viral contig excluded from vConTACT2 due to >99% similarity to the entry directly above it in the table.

** Viral contigs identified by blastp but excluded from phage search due to contig lengths.

[†] Viral contig encodes VqmAφ-Qtip but does not cluster with VP882-like phages.

bioRxiv preprint doi: https://doi.org/10.1101/2025.07.30.667625; this version posted July 30, 2025. The copyright holder for this preprint (which was not certified by peer review) is the author/funder, who has granted bioRxiv a license to display the preprint in perpetuity. It is made available under aCC-BY 4.0 International license.

Table S2. Strains and plasmids used in this study.

Bacterial Strains<br>Parent Strain	Identifier	Genotype	Reference
V. cholerae C6706	BB-Vc0325	Δ*tdh* Δ*lacZ*::P$_{vqmR}$-*luxCDABE*	(2)
BB-Vc0325	FJS-S1644	/pXBCm-P$_{bad\text{-}riboswitch}$-*gfp*	This study
	FJS-S1645	/pXBCm-P$_{bad\text{-}riboswitch}$-*tdh*^{Vp}	This study
	FJS-S1648	/pXBCm-P$_{bad\text{-}riboswitch}$-*tdh*^{Vv}	This study
	FJS-S1649	/pXBCm-P$_{bad\text{-}riboswitch}$-*tdh*^{Vc}	This study
	FJS-S1650	/pXBCm-P$_{bad\text{-}riboswitch}$-*tdh*^{Sa}	This study
	FJS-S1651	/pXBCm-P$_{bad\text{-}riboswitch}$-*tdh*^{Se1}	This study
	FJS-S1652	/pXBCm-P$_{bad\text{-}riboswitch}$-*tdh*^{Se2}	This study
	FJS-S1653	/pXBCm-P$_{bad\text{-}riboswitch}$-*tdh*^{C6706}	This study
V. parahaemolyticus RIMD 2210633	FJS-S0063	Δ*tdh*; Amp^{R}, Sm^{R}	This study
FJS-S0063	FJS-S1614	/pEVS143-P$_{qtip}$-*luxCDABE*<br>/pXBCm-P$_{bad\text{-}riboswitch}$-*gfp*	This study
	FJS-S1615	/pEVS143-P$_{qtip}$-*luxCDABE*<br>/pXBCm-P$_{bad\text{-}riboswitch}$-*vqmA*φ^{1}	This study
	FJS-S1655	/pEVS143-P$_{qtip}$-*luxCDABE*<br>/pXBCm-P$_{bad\text{-}riboswitch}$-*vqmA*φ^{2}	This study
	FJS-S1656	/pEVS143-P$_{qtip}$-*luxCDABE*<br>/pXBCm-P$_{bad\text{-}riboswitch}$-*vqmA*φ^{3}	This study
	FJS-S1657	/pEVS143-P$_{qtip}$-*luxCDABE*<br>/pXBCm-P$_{bad\text{-}riboswitch}$-*vqmA*φ^{4}	This study
	FJS-S1658	/pEVS143-P$_{qtip}$-*luxCDABE*<br>/pXBCm-P$_{bad\text{-}riboswitch}$-*vqmA*φ^{5}	This study
	FJS-S1659	/pEVS143-P$_{qtip}$-*luxCDABE*<br>/pXBCm-P$_{bad\text{-}riboswitch}$-*vqmA*φ^{6}	This study
E. coli TOP10		*F– mcrA Δ(mrr-hsdRMS-mcrBC) φ80lacZΔM15 ΔlacX74 recA1 araD139 Δ(ara-leu)7697 galU galK λ– rpsL(Str*^{R}*) endA1 nupG*	Invitrogen
**Plasmids**<br>**Name**	**Identifier**	**Description**	**Reference**
pXBCm-P$_{bad\text{-}riboswitch}$-*gfp*	FJS-P122	Dual-control expression construct (arabinose-inducible transcription/theophylline-inducible translation) on a chloramphenicol-resistant version of the pXB300 (3) backbone; ColE1; Cm^{R}	This study
pXBCm-P$_{bad\text{-}riboswitch}$-*tdh*^{Vp}	FJS-P127	*Vibrio parahaemolyticus* 882 *tdh* allele expression plasmid; ColE1; Cm^{R}	This study

bioRxiv preprint doi: https://doi.org/10.1101/2025.07.30.667625; this version posted July 30, 2025. The copyright holder for this preprint (which was not certified by peer review) is the author/funder, who has granted bioRxiv a license to display the preprint in perpetuity. It is made available under aCC-BY 4.0 International license.

pXBCm-$P_{bad\text{-}riboswitch}$-*tdh*^{Vv}	FJS-P131	*Vibrio vulnificus* CUVETCC1 *tdh* allele expression plasmid; ColE1; Cm^R	This study
pXBCm-$P_{bad\text{-}riboswitch}$-*tdh*^{Vc}	FJS-P132	*Vibrio cholerae* 497778 *tdh* allele expression plasmid; ColE1; Cm^R	This study
pXBCm-$P_{bad\text{-}riboswitch}$-*tdh*^{Sa}	FJS-P133	*Shewanella algae* CLS1 *tdh* allele expression plasmid; ColE1; Cm^R	This study
pXBCm-$P_{bad\text{-}riboswitch}$-*tdh*^{Se1}	FJS-P134	*Salmonella enterica* PNUSAS042767 *tdh* allele expression plasmid; ColE1; Cm^R	This study
pXBCm-$P_{bad\text{-}riboswitch}$-*tdh*^{Se2}	FJS-P135	*Salmonella enterica* PNUSAS361348 *tdh* allele expression plasmid; ColE1; Cm^R	This study
pXBCm-$P_{bad\text{-}riboswitch}$-*tdh*^{C6706}	FJS-P136	*Vibrio cholerae* C6706 *tdh* allele expression plasmid; ColE1; Cm^R	This study
pEVS143-P_{qtip}-*luxCDABE*	pOD-58	Transcriptional fusion of the φVP882 *qtip* promoter to the *luxCDABE* operon; p15A; Km^R	(4)
pXBCm-$P_{bad\text{-}riboswitch}$-*vqmAφ*^{1}	FJS-P128	*Vibrio parahaemolyticus* 882 *vqmAφ* allele expression plasmid; ColE1; Cm^R	This study
pXBCm-$P_{bad\text{-}riboswitch}$-*vqmAφ*^{2}	FJS-P138	*Vibrio parahaemolyticus* 1171-97 *vqmAφ* allele expression plasmid; ColE1; Cm^R	This study
pXBCm-$P_{bad\text{-}riboswitch}$-*vqmAφ*^{3}	FJS-P139	*Vibrio parahaemolyticus* VP13206 *vqmAφ* allele expression plasmid; ColE1; Cm^R	This study
pXBCm-$P_{bad\text{-}riboswitch}$-*vqmAφ*^{4}	FJS-P140	*Salmonella enterica* PNUSAS042767 *vqmAφ* allele expression plasmid; ColE1; Cm^R	This study
pXBCm-$P_{bad\text{-}riboswitch}$-*vqmAφ*^{5}	FJS-P141	*Vibrio parahaemolyticus* VP10429 *vqmAφ* allele expression plasmid; ColE1; Cm^R	This study
pXBCm-$P_{bad\text{-}riboswitch}$-*vqmAφ*^{6}	FJS-P142	*Rikenellaceae* bacterium MAG302 *vqmAφ* allele expression plasmid; ColE1; Cm^R	This study

bioRxiv preprint doi: https://doi.org/10.1101/2025.07.30.667625; this version posted July 30, 2025. The copyright holder for this preprint (which was not certified by peer review) is the author/funder, who has granted bioRxiv a license to display the preprint in perpetuity. It is made available under aCC-BY 4.0 International license.

Table S3. Primers and synthetic DNA fragments used in this study.

Primers Identifier	Sequence (5'→3')*	Description / Associated Construct
FJS-O549	ATGAGAGAAGATTTTCAGCCTG	Forward primer to linearize the pXBCm backbone without the *tetR*-P_{tetA} machinery / pXBCm-$P_{bad\text{-}riboswitch}$-*gfp*
FJS-O550	GCTTCAGTAGTCAGACCAG	Reverse primer to linearize the pXBCm backbone without the *tetR*-P_{tetA} machinery / pXBCm-$P_{bad\text{-}riboswitch}$-*gfp*
FJS-O551	caggactgatgctggtctgactactgaagcCTATGCTACTCCGTCAAGC	Forward primer for $P_{bad\text{-}riboswitch}$ insert from strain TND2292 (5) / pXBCm-$P_{bad\text{-}riboswitch}$-*gfp*
FJS-O552	atctgtatcaggctgaaaatcttctctcatCAAATAAAGCCAGTACAAACTG	Reverse primer for $P_{bad\text{-}riboswitch}$ insert from strain TND2292 (5) / pXBCm-$P_{bad\text{-}riboswitch}$-*gfp*
FJS-O555	GTTGAGTTGGATGCAGCACC	Forward primer to linearize the pXBCm-$P_{bad\text{-}riboswitch}$-*gfp* backbone without the *gfp* gene / all pXBCm-$P_{bad\text{-}riboswitch}$ constructs
FJS-O556	CTTGTTGTTACCTCCTTAGCAGG	Reverse primer to linearize the pXBCm-$P_{bad\text{-}riboswitch}$-*gfp* backbone without the *gfp* gene / all pXBCm-$P_{bad\text{-}riboswitch}$ constructs
FJS-O561	gcagcaccctgctaaggaggtaacaacaagATGAAAATTAAAGCACTATCAAAGCT	Forward primer for *tdh*^{Vp} insert from *V. parahaemolyticus* strain 882 / pXBCm-$P_{bad\text{-}riboswitch}$-*tdh*^{Vp}
FJS-O562	agcaatttatggtgctgcatccaactcaacCTATTCCCAATCAAGAATAACTTTGCC	Reverse primer for *tdh*^{Vp} insert from *V. parahaemolyticus* strain 882 / pXBCm-$P_{bad\text{-}riboswitch}$-*tdh*^{Vp}
FJS-O571	gcagcaccctgctaaggaggtaacaacaagATGGAAATCAAAGCACTTTCA	Forward primer for *tdh*^{C6706} insert from pKP-443 (6) / pXBCm-$P_{bad\text{-}riboswitch}$-*tdh*^{C6706}

bioRxiv preprint doi: https://doi.org/10.1101/2025.07.30.667625; this version posted July 30, 2025. The copyright holder for this preprint (which was not certified by peer review) is the author/funder, who has granted bioRxiv a license to display the preprint in perpetuity. It is made available under aCC-BY 4.0 International license.

FJS-O572	agcaatttatggtgctgcatccaactcaacCTACTGCCAATCGAGGATAACTTTGC	Reverse primer for tdh^{C6706} insert from pKP-443 (6) / pXBCm-$P_{bad\text{-}riboswitch}$-$tdh^{C6706}$
FJS-O573	gcagcaccctgctaaggaggtaacaacaagATGTCAATAAGCGAAGGGGATGA	Forward primer for $vqmA\varphi^{1}$ insert from *V. parahaemolyticus* strain 882 / pXBCm-$P_{bad\text{-}riboswitch}$-$vqmA\varphi^{1}$
FJS-O574	agcaatttatggtgctgcatccaactcaacCTACTTGAGCAGCATCGAGAC	Reverse primer for $vqmA\varphi^{1}$ insert from *V. parahaemolyticus* strain 882 / pXBCm-$P_{bad\text{-}riboswitch}$-$vqmA\varphi^{1}$
**Fragments Name**	**Sequence (5'→3')***	**Description / Associated Construct**
tdh^{Vv}	gcagcaccctgctaaggaggtaacaacaagATGAAAATCAAAGCACTATCAAAGCTAAAGCCAGAAGAAGGCATTTGGATGACCGAAGTGGACAAGCCTGTTCTTGGCCACAACGATCTGCTGATCAAAATTAAGAAAACCGCGATTTGTGGTACCGACGTACACATCTACAACTGGGACGAATGGTCACAAAAAACCATCCCAGTACCTATGGTGGTTGGCCATGAATACGTGGGTGAAGTGGTTGGCATTGGCCAAGAAGTTCGTGGTTTTGAGATCGGTGACCGCGTTTCTGGCGAAGGTCACATCACTTGTGGCCACTGTCGTAACTGCCGTGGTGGCCGCACGCACTTGTGCCGCAACACCATTGGTGTGGGCGTAAACCGCACAGGTTGTTTCTCTGAATACCTTGTGATCCCAGCGTTCAACGCCTTTAAAATCCCTGCAAACATCTCTGATGATCTTGCGTCTATCTTCGACCCGTTTGGCAACGCAGTACACACAGCACTGTCGTTCGATCTGGTTGGTGAAGACGTACTGATCACCGGCGCTGGCCCAATCGGCATCATGGCGGCTGCGGTAGCGAAGCACGTTGGTGCGCGCCACGTCGTGATCACCGATGTGAACGAATACCGTCTAGACCTCGCACGCAAAATGGGCGTGACTCGCGCGGTGAACGTTGCCGAGCAGAAGCTTGACGATGTGATGGCAGAGCTAGGCATGACAGAAGGCTTCGATGTGGGCCTGGAAATGTCGGGCAACCCATCAGCATTCAACTCAATGCTGAAAACCATGAACCACGGTGGCCGTATTGCACTGCTTGGCATTCCACCATCAGACATGGGCATCGATTGGAACCAAGTGATCTTCAAAGGCTTGGTGATTAAAGGTATCTATGGTCGTGAAATGTTTGAAACTTGGTACAAGATGGCGAGCTTGATTCAATCTGGCCTTGACCTAACACCAATTATCACTCACCACTTCAAAGTGGATGATTTCCAGCAAGGCTTCGACATCATGCGCAGCGGCATGTCAGGCAAAGTGATTCTTGATTGGGAATAAgttgagttggatgcagcaccataaattgct	Synthetic insert sequence for tdh^{Vv} from *V. vulnificus* strain CUVETCC1 / pXBCm-$P_{bad\text{-}riboswitch}$-$tdh^{Vv}$
tdh^{Vc}	gcagcaccctgctaaggaggtaacaacaagATGAAAATCAAAGCACTTTCAAAACTGAAACCAGAGCAGGGCATCTGGATGAACGAAGTGGACATGCCTGAGCTTGGCCACAACGACCTGCTGATCAAAATTAAGAAAACCGCCATTTGTGGTACTGACGTACACATTTATAACTGGGATGAGTGGTCACAAAAAACCATTCCAGTGCCTATGGTAGTCGGCCATGAATATGTGGGTGAAGTGGTTGGGATTGGCCAAGAAGTGCGTGGTTTCCAAATTGGTGATCGCGTTTCTGGCGAAGGTCACATCACTTGTGGTCACTGCCGTAACTGCCGTGGCGGCCGTACGCACCTGTGCCGTAACACCATTGGTGTGGGCGTAAACCGCACGGGTTGTTTTTCTGAATACTTAGTGATCCCAGCGTTTAACGCATTCAAGATCCCGGATGGTATTTCAGATGATCTGGCGTCTATCTTCGACCCGTTTGGAAACGCTGTACACACCGCGCTTTCATTCGACTTAGTGGGTGAAGATGTTCTGATCACCGGTGCTGGCCCAATCGGCATTATGGCCGCTGCGGTTGCAAAACACGTTGGTGCGCGCCATGTGGTGATCACTGATGTGAACGAATACCGCCTCGATTTAGCTCGTAAAATGGGTGTGACTCGCGC	Synthetic insert sequence for tdh^{Vc} from *V. cholerae* strain 497778 / pXBCm-$P_{bad\text{-}riboswitch}$-$tdh^{Vc}$

bioRxiv preprint doi: https://doi.org/10.1101/2025.07.30.667625; this version posted July 30, 2025. The copyright holder for this preprint (which was not certified by peer review) is the author/funder, who has granted bioRxiv a license to display the preprint in perpetuity. It is made available under aCC-BY 4.0 International license.

	TGTGAACGTTGCGGAGCAAAATCTAGAAGATGTGATGAAAGAGC<br>TCGGCATGACCGAAGGTTTTGATGTGGGCTTAGAGATGTCTGGC<br>GTACCGAGTGCGTTTAGCGCCATGCTAAAAACCATGAACCATGG<br>TGGCCGCATCGCTCTGTTAGGTATTCCACCTTCATCGATGGCGAT<br>TGATTGGAACCAGGTGATCTTCAAAGGCCTTGTTATTAAAGGGAT<br>TTATGGAAGGGAAATGTTCGAAACTTGGTATAAGATGGCGAGCCT<br>CATTCAATCGGGTCTCGATATCAGCCCAATTATCACTCACCACTTC<br>AAAGTGGATGACTTCCAAAAAGGCTTCGACATCATGCGCAGCGG<br>GGCTTCCGGCAAAGTTATCCTCGATTGGCAGTAAgttgagttggatgcag<br>caccataaattgct	
tdh^{Sa}	gcagcaccctgctaaggaggtaacaacaagATGAAAGCACTGAGTAAACTCA<br>AGCCTGAACAAGGCATCTGGATGGTAGACGCGCCCAAACCTGAA<br>ATGGGCCATAACGATCTGCTGATCAAGATTCGCAAGACCGCCATT<br>TGTGGTACCGATGTGCATATCTACAACTGGGATGAGTGGTCACAA<br>AAGACCATCCCGGTTCCTATGGTTGTCGGCCATGAATATGTCGG<br>CGAAGTGGTAGATATGGGTCAGGAAGTTCGTGGCTTTAATATTGG<br>TGACCGAGTGTCAGGCGAAGGTCATATCACCTGTGGTCACTGCC<br>GTAATTGCCGTGGTGGCCGCACTCATTTGTGCCGTAACACTGTG<br>GGTGTTGGGGTTAACCGCGAAGGCGCCTTCGCCGAATATCTGGT<br>GATCCCGGCCTTCAACGCCTTCAAGATCCCCGATGATATCAGCG<br>ACGATCTGGCCGCTATCTTCGACCCCTTTGGCAACGCGGTACAC<br>ACTGCGCTGTCGTTTGATCTCGTCGGTGAAGATGTGCTGATCAC<br>CGGAGCCGGCCCCATAGGCATTATGGCCGCCGCGGTTTGCCGC<br>CATGTCGGTGCCCGCCACGTAGTTGTCACAGATGTCAACGAATA<br>CCGTCTAGAGCTGGCGCGCAAGCTGGGAGCGACCCGAGCCGT<br>CAATGTGGCCAAGGAAAAACTCGAAGATGTGATGAGTGAGCTCG<br>GCATGACAGAGGGCTTCGATGTCGGCCTGGAAATGTCCGGTGTA<br>CCGTCAGCCTTCCACTCCATGTTGGATACCATGAACCACGGTGG<br>CAAAATTGCCATGTTGGGGATCCCGGGTGGCGATATGGCGATAG<br>ACTGGAGCAAGGTCATCTTCAAGGGGTTGGTGATAAAGGGCATC<br>TATGGCCGGGAAATGTTTGAAACCTGGTACAAGATGGCCAGTTT<br>GATCCAGTCCGGATTGGATATTGCGCCTATTATCACCCACCATTAC<br>AAGGTGGATGACTTCCAGCAGGGCTTCGATGCCATGCGCTCGG<br>GTCAATCCGGCAAGGTCATTCTCAGCTGGGATTGAgttgagttggatgc<br>agcaccataaattgct	Synthetic insert sequence for *tdh^{Sa}* from *S. algae* strain CLS1 / pXBCm-$P_{bad\text{-}riboswitch}$-*$tdh^{Sa}$*
tdh^{Se1}	gcagcaccctgctaaggaggtaacaacaagATGAAAGCGTTATCCAAACTGA<br>AAGCGGAAGAGGGCATCTGGATGACCGACGTTCCGGAACCGGA<br>AGTCGGCCATAACGATTTGCTGATTAAAATCCGTAAAACAGCCAT<br>CTGCGGCACTGACGTTCACATCTATAACTGGGATGACTGGTCGC<br>AAAAAACCATCCCGGTTCCGATGGTCGTGGGGCATGAATATGTC<br>GGCGAAGTGGTCGGCATCGGTCAGGAAGTGAAAGGCTTTAAAAT<br>CGGCGATCGCGTCTCCGGCGAAGGTCACATCACCTGTGGTCATT<br>GTCGCAACTGCCGTGGCGGTCGTACTCACCTGTGCCGCAACAC<br>CACCGGCGTAGGCGTTAATCGTCCCGGCTGCTTCGCGGAATACC<br>TGGTCATCCCGGCATTCAATGCGTTTAAAATCCCGGATAACATTT<br>CTGATGATTTAGCCTCTATTTTCGACCCGTTTGGTAATGCGGTGC<br>ATACGGCGCTGTCTTTCGATCTGGTCGGCGAAGATGTACTGGTAT<br>CGGGGGCGGGGCCAATCGGCGTAATGGCCGCCGCGGTGGCGA<br>AACATGTTGGCGCGCGTCATGTGGTGATTACTGACGTCAATGAAT<br>ACCGTCTGGAGCTGGCGCGCAAAATGGGCGTCACCCGCGCGG<br>TCAACGTCGCGAAAGAGAGCCTGAACGACGTCATGGCGGAGCT<br>GGGAATGACCGAAGGGTTCGATGTGGGTCTGGAGATGTCCGGC<br>GCGCCGCCGGCGTTTCGTACCATGCTGGACACCATGAATCACG<br>GCGGTCGTATTGCGATGCTGGGGATTCCGCCATCAGATATGTCTA<br>TCGACTGGACAAAAGTCATCTTTAAAGGCTTGTTCATTAAAGGTAT<br>TTATGGTCGTGAGATGTTCGAAACGTGGTACAAAATGGCGGCGC<br>TGATCCAGTCCGGTCTGGATCTGTCACCGATTATCACCCATCGTT<br>TCTCTATTGATGATTTCCAGAAAGGTTTTGATGCCATGCGTTCAG<br>GCCAGTCAGGAAAAGTTATTCTGAGCTGGGATTAAgttgagttggatgc<br>agcaccataaattgct	Synthetic insert sequence for *tdh^{Se1}* from *S. enterica* strain PNUSAS042767 / pXBCm-$P_{bad\text{-}riboswitch}$-*$tdh^{Se1}$*

bioRxiv preprint doi: https://doi.org/10.1101/2025.07.30.667625; this version posted July 30, 2025. The copyright holder for this preprint (which was not certified by peer review) is the author/funder, who has granted bioRxiv a license to display the preprint in perpetuity. It is made available under aCC-BY 4.0 International license.

tdh^{Se2}	gcagcaccctgctaaggaggtaacaacaagATGAAAGCGTTATCCAAACTGA<br>AAGCGGAAGAGGGCATCTGGATGACCGACGTTCCGGAACCGGA<br>AGTCGGCCATAACGATTTGCTGATTAAAATCCGTAAAACAGCCAT<br>CTGCGGTACTGACGTTCACATCTATAACTGGGATGACTGGTCGC<br>AAAAAACCATCCCGGTTCCGATGGTCGTGGGGCATGAATATGTT<br>GGCGAAGTGGTCGGCATCGGTCAGGAAGTGAAAGGCTTTAAAAT<br>TGGCGATCGCGTCTCCGGCGAAGGTCATATCACCTGTGGTCATT<br>GCCGCAACTGCCGTGGTGGTCGTACTCACCTGTGTCGCAACAC<br>CACCGGCGTGGGCGTCAACCGTCCCGGCTGCTTCGCGGAATAT<br>CTGGTCATCCCGGCGTTCAATGCGTTTAAAATCCCGGATAACATT<br>TCTGATGATTTAGCCTCTATTTTCGACCCGTTTGGTAATGCGGTG<br>CATACGGCGCTGTCTTTCGATCTGGTCGGCGAAGATGTACTGGT<br>ATCGGGGGCGGGGCCAATCGGCGTAATGGCCGCCGCGGTGGC<br>GAAACATGTTGGCGCGCGTCATGTGGTGATTACTGACGTCAATG<br>AATACCGTCTGGAGCTGGCGCGCAAAATGGGCGTCACCCGCGC<br>GGTCAACGTCGCGAAAGAGAGCCTGAACGACGTCATGGAGGAG<br>CTGGGAATGACCGAAGGATTCGATGTGGGTCTGGAGATGTCCG<br>GCGCGCCGCCGGCGTTTCGTACCATGCTGGACACCATGAATCA<br>CGGCGGTCGTATTGCGATGCTGGGGATTCCGCCATCAGATATGT<br>CTATCGACTGGACAAAAGTTATCTTTAAGGGCTTGTTCATTAAAG<br>GTATTTATGGTCGTGAGATGTTCGAAACGTGGTACAAAATGGCGG<br>CGCTGATCCAGTCCGGTCTGGATCTGTCACCGATTATCACCCATC<br>GTTTCTCTATTGATGATTTCCAGAAAGGTTTTGACGCCATGCGTT<br>CAGGCCAGTCAGGAAAAGTTATTCTGAGCTGGGATTAAgttgagttgg<br>atgcagcaccataaattgct	Synthetic insert sequence for tdh^{Se2} from *S. enterica* strain PNUSAS361348 / pXBCm-$P_{bad-riboswitch}$-tdh^{Se2}
$vqmA\varphi^2$	gcagcaccctgctaaggaggtaacaacaagATGTCAATAAGCGAAGGGGATG<br>ATGCTTACATCCGCTCGTTGATTCATTTTTTTGGCAATCAACCGGA<br>TCCGTGGGGCATCAAGGACACCAAGTCGGTGTTCATCTATGCAA<br>ACCAGCCCTTTCGAGAGTTAGTCGGTATGAAGAACCGCAACGTG<br>GAAGGACTTACCGACGCTGATATGGATTGCGAAACTGCGGCCTT<br>TGCCGACTCCTTTCAGGCCCAAGATAGGCTGGTCGAGCAAGGC<br>CGGGAGAAGAAAATCGTCCTGGACGTACACCCCTACGCGAATG<br>GTTGGCGCGTTTTCACTTTCACCAAGACCCCTCTCATCATGCCG<br>TCCGGACGTGTGGCCGGCACCATTTTCCACGGACAAGACCTGA<br>CTGACACGGCTGGCCGCATCGAGCCTGCAGTGGTTGAGCTGCT<br>GCTGCCTTCCAGTGGCCAGGCTGGATCCTTCGAGACCAATGTG<br>GTCGGTCTCAACTTGACCGAACGCGAGGAACTGGTGCTGTTCTT<br>CCTGCTTCGTGGCCGAACGGCCAAGGATATCGCTGGCATGCTG<br>GGGCGCTCTCCCCGCACCATCGAACACGCTATCGAGCGCATCC<br>GCAACAAATTCGGTGCTGGCAACAAGCGGGAGCTCATCGATATG<br>GCCATGTCCAAGGGTTATTACAGCATGGTGCCAAAAGCCCTGTTT<br>CACACACAGGTCTCGATGCTGCTCAAGTAGgttgagttggatgcagcacc<br>ataaattgct	Synthetic insert sequence for $vqmA\varphi^2$ from *V. parahaemolyticus* strain 1171-97 / pXBCm-$P_{bad-riboswitch}$-$vqmA\varphi^2$
$vqmA\varphi^3$	gcagcaccctgctaaggaggtaacaacaagATGTCAATAAGCGAAGGGGATG<br>ATGCTTACATCCGCTCGTTGATTCATTTTTTTGGCAATCAACCGGA<br>TCCGTGGGGCATCAAGGACACCAAGTCGGTGTTCATCTATGCAA<br>ACCAGCCCTTTCGAGAGTTAGTCGGTATGAAGAACCGCAATGTG<br>GAAGGACTTACCGACGCCGATATGGATTGCGAAACTGCGGCCTT<br>TGCCGACTCCTTTCAGGCCCAAGATAGGCTGGTCGAGCAAGGC<br>CGGGAGAAGAAAATCGTCCTGGACGTACACCCCTACGCGAATG<br>GTTGGCGCGTTTTCACGTTCACCAAGACCCCTCTCATCATGCCG<br>TCCGGACGTGTGGCCGGCACCATTTTCCACGGACAAGACCTGA<br>CTGACACTGCTGGCCGCATCGAGCGTGCAGTGGTTGACCTGCT<br>GCTGCCTTCCAGTGGCCAGGCTGGATCCTTCGAGACCAATGTG<br>GTCGGTCTCAACTTGACCGAACGCGAGGAGCTGGTGCTGTTCT<br>TCCTGCTTCGTGGCAGAACAGCCAAGGATATCGCTGGCATGCTG<br>GGGCGCTCTCCCCGCACCATCGAACACGCTATCGAGCGCATCC<br>GCAACAAATTCGGTGCTGGCAACAAGCGGGAGCTCATCGATATG<br>GCCATGTCCAAGGGTTATTACACCATGGTGCCAAAAGCCCTGTTT<br>CACACACAGGTCTCGATGCTGCTCAAGTAGgttgagttggatgcagcacc<br>ataaattgct	Synthetic insert sequence for $vqmA\varphi^3$ from *V. parahaemolyticus* strain VP13206 / pXBCm-$P_{bad-riboswitch}$-$vqmA\varphi^3$

bioRxiv preprint doi: https://doi.org/10.1101/2025.07.30.667625; this version posted July 30, 2025. The copyright holder for this preprint (which was not certified by peer review) is the author/funder, who has granted bioRxiv a license to display the preprint in perpetuity. It is made available under aCC-BY 4.0 International license.

vqmAφ[4]	gcagcaccctgctaaggaggtaacaacaagATGTCAATAAGCGAAGGGGATG<br>ATGCTTACATCCGCTCGTTGATTCATTTTTTTGGCAATCAACCGGA<br>TCCGTGGGGCATCAAGGACACCAAGTCGGTGTTCATCTATGCAA<br>ACCAGCCCTTTCGAGAGTTAGTCGGTATGAAGAACCGCAATGTG<br>GAAGGACTTACCGACGCCGATATGGATTGCGAAACTGCGGCCTT<br>TGCCGACTCCTTTCAGGCCCAAGATAGGCTGGTCGAGCAAGGC<br>CGGGAGAAGAAAATCGTCCTGGACGTACACCCCTACGCGAATG<br>GTTGGCGCGTTTTCACGTTCACCAAGACCCCTCTCATCATGCCG<br>TCCGGACGTGTGGCCGGCACCATTTTCCACGGACAAGACCTGA<br>CTGACACGGCTGGCCGCATCGAGCGTGCAGTGGTTGAGCTGCT<br>GCTGCCTTCCAGTGGCCAGGCTGGATCCTTCGAGACCAATGTG<br>GTCGGTCTCAACTTGACCGAACGCGAGGAGCTGGTGCTGTTCT<br>TCCTGCTTCGTGGCCGAACGGACAAGGATATCGCTGGCATGCTG<br>GGGCGCTCTCCCCGCACCATCGAACACGCTATCGAGCGCATCC<br>GCAACAAATTCGGTGCTGGCAACAAGCGGGAGCTCATCGATATG<br>GCCATGTCCAAGGGTTATTACACCATGGTACCAAAAGCCCTGTTT<br>CACACACAGGTCTCGATGCTGCTCAAGTAGgttgagttggatgcagcacc<br>ataaattgct	Synthetic insert sequence for *vqmAφ*[4] from *S. enterica* strain PNUSAS042767 / pXBCm-$P_{bad\text{-}riboswitch}$-*vqmAφ*[4]
vqmAφ[5]	gcagcaccctgctaaggaggtaacaacaagATGTCAATAAGCGAAGGGGATG<br>ATGCTTACATCCGCTCGTTGATTCATTTTTTTGGCAATCAACCGGA<br>TCCGTGGGGCATCAAGGACACCAAGTCGGTGTTCATCTATGCAA<br>ACCAGCCCTTTCGAGAGTTAGTCGGTATGAAGAACCGCAATGTG<br>GAAGGACTTACCGACGCCGATATGGATTGCGAAACTGCGGCCTT<br>TGCCGACTCCTTTCAGGCCCAAGATAGGCTGGTCGAGCAAGGC<br>CGGGAGAAGAAAATCGTCCTGGACGTACACCCCTACGCGAATG<br>GTTGGCGCGTTTTCACGTTCACCAAGACCCCTCTCATCATGCCG<br>TCCGGACGTGTGGCCGGCACCATTTTCCACGGACAAGACCTGA<br>CTGACACTGCTGGCCGCATCGAGCGTGCAGTGGTTGACCTGCT<br>GCTGCCTTCCAGTGGCCAGGCTGGATCCTTCGAGACCAATGTG<br>GTCGGTCTCAACTTGACCGAACGCGAGGAGCTGGTGCTGTTCT<br>TCCTGCTTCGTGGCAGAACAGCCAAGGATATCGCAGGCATGCTG<br>GGGCGCTCTCCCCGCACCATCGAACACGCTATCGATCGCATCCG<br>CAACAAATTCGGTGCTGGCAACAAGCGGGAGCTCATCGATATGG<br>CCATGTCCAAGGGTTATTACACCATGGTGCCAAAAGCCCTGTTTC<br>ACACACAGGTCTCGATGCTGCTCAAGTAGgttgagttggatgcagcaccat<br>aaattgct	Synthetic insert sequence for *vqmAφ*[5] from *V. parahaemolyticus* strain VP10429/ pXBCm-$P_{bad\text{-}riboswitch}$-*vqmAφ*[5]
vqmAφ[6]	gcagcaccctgctaaggaggtaacaacaagATGTCAATAAGCGAAGGGGATG<br>ATGCTTACATCCGCTCGTTGATTCATTTTTTTGGCAATCAACCGGA<br>TCCGTGGGGCATCAAGGACACCAAGTCGGTGTTCATCTATGCAA<br>ACCAGCCCTTTCGAGAGTTAGTCGGTATGAAGAACCGCAATGTG<br>GATTGCGAAACTGCGGCCTTTGCCGACTCCTTTCAGGCCCAAGA<br>TAGGCTGGTCGAGCAAGGCCGGGAGAAGAAAATCGTCCTGGAC<br>GTACACCCCTACGCGAATGGTTGGCGCGTTTTCACTTTCACCAA<br>GACCCCTCTCATCATGCCGTCCGGACGTGTGGCCGGCACCATTT<br>TTCACGGACAAGACCTGACTGACACGGCTGGCCGCATCGAGCG<br>TGCAGTGGTTGACCTGCTGCTGCCTTCCAGTGGCCAGGCTGGA<br>TCCTTCGAGACCAATGTGGTCGGTCTCAACTTGACTGAACGCGA<br>GGAGCTGGTGCTGTTCTTCCTGCTTCGTGGCCGAACGGCCAAG<br>GATATCGCTGGCATGCTGGGGCGCTCTCCCCGCACCATCGAACA<br>CGCTATCGAGCGCATCCGCAACAAATTCGGTGCTGGCAACAAGC<br>GGGAGCTCATCGATATGGCCATGTCCAAGGGTTATTACACCATGG<br>TGCCAAAAGCCCTGTTTCACACACAGGTCTCGATGCTTCTCAAG<br>TAGgttgagttggatgcagcaccataaattgct	Synthetic insert sequence for *vqmAφ*[6] from *Rikenellaceae* MAG302 / pXBCm-$P_{bad\text{-}riboswitch}$-*vqmAφ*[6]

* Lowercase letters represent homologous overlaps for Fast Cloning.

bioRxiv preprint doi: https://doi.org/10.1101/2025.07.30.667625; this version posted July 30, 2025. The copyright holder for this preprint (which was not certified by peer review) is the author/funder, who has granted bioRxiv a license to display the preprint in perpetuity. It is made available under aCC-BY 4.0 International license.

SUPPLEMENTAL REFERENCES

1. Lan S-F, Huang C-H, Chang C-H, Liao W-C, Lin I-H, Jian W-N, Wu Y-G, Chen S-Y, Wong H. 2009. Characterization of a New Plasmid-Like Prophage in a Pandemic *Vibrio parahaemolyticus* O3:K6 Strain. *Appl Environ Microbiol* 75:2659–2667.

2. Mashruwala AA, Bassler BL. 2020. The *Vibrio cholerae* Quorum-Sensing Protein VqmA Integrates Cell Density, Environmental, and Host-Derived Cues into the Control of Virulence. *mBio* 11:10.1128/mbio.01572-20.

3. Bina XR, Wong EA, Bina TF, Bina JE. 2014. Construction of a tetracycline inducible expression vector and characterization of its use in *Vibrio cholerae*. *Plasmid* 76:87–94.

4. Duddy OP, Silpe JE, Fei C, Bassler BL. 2023. Natural silencing of quorum-sensing activity protects *Vibrio parahaemolyticus* from lysis by an autoinducer-detecting phage. *PLoS Genet* 19:e1010809.

5. Dalia TN, Chlebek JL, Dalia AB. 2020. A modular chromosomally integrated toolkit for ectopic gene expression in *Vibrio cholerae*. *Sci Rep* 10:15398.

6. Papenfort K, Silpe JE, Schramma KR, Cong J-P, Seyedsayamdost MR, Bassler BL. 2017. A *Vibrio cholerae* autoinducer–receptor pair that controls biofilm formation. *Nat Chem Biol* 13:551–557.
